# Electrospinning Evolution Derived from TRIZ Theory for Directly Writing Patterned Nanofibers

**DOI:** 10.3390/polym15143091

**Published:** 2023-07-19

**Authors:** Yuchao Wu, Zhanghong Liu, Hongtao Wu, Kai Zhang, Qingjie Liu

**Affiliations:** 1 Aircraft Manufacturing School of Sichuan Aerospace Vocational College, Chengdu 610100, China; schtxy666wyc@163.com (Y.W.); liuzhanghong@my.swjtu.edu.cn (Z.L.); wuhongtao@mail.swust.edu.cn (H.W.); 2 School of Mechanical Engineering, Sichuan University, Chengdu 610065, China; zhangkai@scu.edu.cn; 3 Yibin Industrial Technology Research Institute of Sichuan University, Yibin 644000, China

**Keywords:** electrospinning, TRIZ theory, charge neutralize, pattern nanofiber

## Abstract

Nanofibers (NFs) have the advantages of tremendous flexibility, small size and a high surface-to-weight ratio and are widely used in sensors, drug carriers and filters. Patterned NFs have expanded their application fields in tissue engineering and electronics. Electrospinning (ES) is widely used to prepare nonwoven NFs by stretching polymer solution jets with electric forces. However, patterned NFs cannot be easily fabricated using ordinary ES methods: the process gradually deteriorates them as repulsion effects between the deposited NFs and the incoming ones increase while residual charges in the fibers accumulate. Repulsion effects are unavoidable because charges in the polymer solution jets are the fundamental forces that are meant to stretch the jets into NFs. TRIZ theory is an effective innovation method for resolving conflicts and eliminating contradictions. Based on the material–field model and the contradiction matrix of TRIZ theory, we propose a strategy to improve ES devices, neutralizing the charges retained in NFs by alternating the current power of the correct frequency, thus successfully fabricating patterned NFs with clear boundaries and good continuity. This study demonstrates a strategy for resolving conflicts in innovation processes based on TRIZ theory and fabricating patterned NFs for potential applications in flexible electronics and wearable sensors.

## 1. Introduction

Nanofibers (NFs) are ultrafine fibers with diameters of less than 1 μm. They have extremely high specific surface areas and porosity and are lightweight and flexible. They occupy an irreplaceable position in composite materials and catalysis [1,2]. Electrospinning (ES), first proposed by Formhals A., is a widely used technique in fabricating NFs with high production efficiency [3]. As bionics and tissue engineering continue to develop, regular-arranged or -patterned NFs are urgently required [4,5]. However, patterned NFs are difficult to obtain using ordinary ES techniques, as whipping instability (also called bending instability) is unavoidable in the spinning process; furthermore, the residual charge’s repulsive effect on the deposited NFs in the collector and the subsequently deposited NFs is a primary obstacle [6].

A few innovative studies have attempted to solve these problems by reducing or eliminating repulsion effects in regular-patterned NFs. For example, Tong et al. tried to solve the problem by utilizing two ES devices, both of them composed of direct current power supplies (DC power), propulsion pumps, spinnerets and collectors. Two DC power supplies were separately connected to the spinnerets, which generated positively and negatively charged polymer solution jets simultaneously and formed NFs that were deposited on individual collectors [7]. As a collector, two rotating cylinders were fixed horizontally on two diagonal quadrants of the disk base, which could periodically rotate back and forth in the horizontal plane; then, those charges attracted to the NFs were neutralized to reduce the repulsion effects in order to prepare thicker 3D fibrous scaffolds, which were much thicker than nonwoven NF mats produced using the traditional ES technique.

Moreover, other researchers have counteracted charge repulsion by introducing external forces. For example, Sun et al. first proved the feasibility of attracting charged NFs from objects with heterogeneous charges using an electrostatic generator, neutralizing the positive residual charges in the NFs. Thus, NFs could form an ideal 3D fiber stack and then be applied to physical devices [8]. Specifically, 2D nonwoven fibrous mats on the collector were connected to the cathode of an electrostatic generator with a copper wire. During the ES process, the electrostatic generator generated electrons in NFs with positive residual charges. As a result, the repulsion effect was notably reduced, and patterned NFs were successfully obtained via layer stacking. These techniques demonstrated effectiveness to some degree; however, they are either complex in structure or expensive.

The theory of inventive problem solving (TRIZ theory) is an effective method for resolving conflicts and eliminating contradictions. Among its techniques, the substance–field model analysis method is used to establish a functional model linked to the problem of an existing system or a new technological system. In the process of problem solving, the corresponding general and standard solutions can be found according to the problem described by the material–field model [9]. To explore innovative attempts, TRIZ theory has been creatively used to improve ES techniques by analyzing the traditional ES system comprehensively, which can help designers work out solutions more systematically and evidentially.

**Figure 1:**
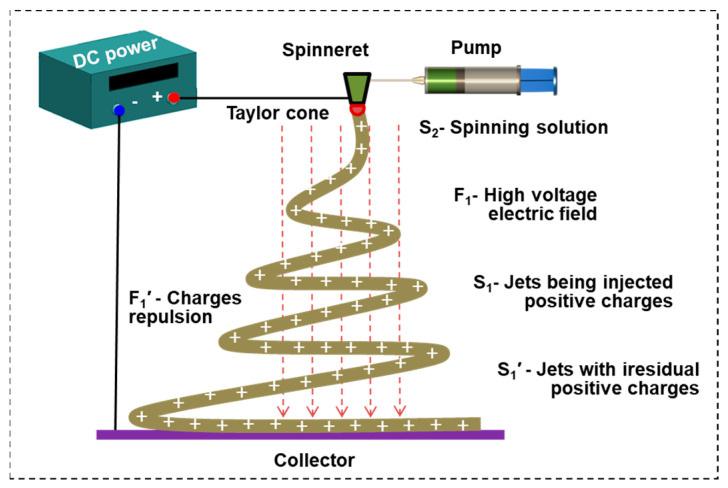
Schematic diagram of charges injected during the ES process.

Usually, ES devices consist of DC power, a syringe pump, a spinneret and a collector [10]. Polymer is dissolved in a solvent and transferred to the syringe pump with a spinneret. The positive electrode is ordinarily connected to the spinneret, and the other one is linked to the collector. An electric field of 1–5 kV/cm is established between the spinneret and the collector, and then, an ES force is formed in the opposite direction toward the liquid; surface tension creates an outward force on the hemisphere-shaped droplet surface [11]. When the electric field gradually increases, the homogenous charges (normally positive) in the solution are forced to gather on the surface of the droplet. The electric field generated by the surface charges of the droplet makes the droplet at the spinneret gradually change from hemispherical to conical (a Taylor cone). When the electric field is large enough, the jet is expelled from the surface of the droplet [12]. In general, the more conductive the solution, the easier it is to form a jet. The jet stream is then accelerated and elongated by the electric field force. Meanwhile, the solvent begins to volatilize, resulting in a polymer jet, and the diameter of the jet decreases to the nanoscale because of solvent volatilization and whipping instability [13]. Finally, polymer-jet-formed NFs are deposited in the collector with residual charges within the NFs. These charges generate repulsive force in incoming NFs and usually produce nonwoven NF mats, as shown in Figure 1.

NF membranes have superhigh surface areas and broad applications. Moreover, patterned fibrous mats with consistent thickness and regular arrangements can expand the current application scenarios and improve their effects, such as in sensors, bionics and biological applications with high performances [14]. However, NFs prepared using ES bear many residual charges. As the same charges repel each other, residual charges attached to fibers collected on the receiving plate will have a repulsive effect on fibers deposited later. It is difficult to prepare patterned fibrous mats with a set thickness, so NFs can only be obtained in a disorderly arrangement. If the residual charges are removed, this is beneficial to preparing patterned fiber mats with regular patterns [15].

## 2. ES Evolution Based on TRIZ Theory

### 2.1. Problem Analysis of ES

TRIZ theory can help designers comprehensively and systematically discuss the creation and realization of technological innovation. TRIZ includes the following principles: ideal final results (IFRs), the 40 inventive principles and substance–field model analysis [16,17]. The corresponding general solutions and standard solutions to invention problems can be obtained based on problems described by the substance–field model [9].

**Figure 2:**
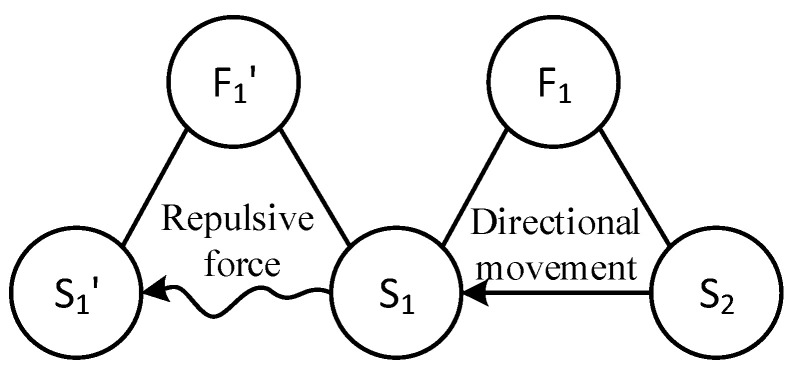
Substance–field model of high-voltage electrospinning.

First, the substance–field model is used here to describe and analyze the problem. After decomposing the high-voltage electrostatic spinning system shown in Figure 1, multiple substance–field triangles can be used for modeling. The process is as follows:

(1) Identification element: The model element can be identified by splitting the high-voltage electrostatic spinning system shown in Figure 1. Substances: S_2_—spinning solution; S_1_—positively charged (+) filament in formation; S_1_′—positively charged (+) filament in formation. Field: F_1_—high voltage electric field; F_1_′—interaction field between electric charges, here shown as repulsion.
(2) Build the model: Based on the organic combination of substances and fields, a substance–field model with two complete triangles is constructed [18], as shown in Figure 2.

Based on an analysis of the substance–field model [19], it can be seen that

(1) The positive charge in the spinning solution, S_2_, moves directionally under the action of the high-pressure electric field, F_1_, forming a positively charged fiber, S_1_ (the effectively complete model).
(2) Under the interaction force, F_1_′, between charges, S_1_ in the fiber being formed has a repulsive effect on S_1_′ of the fiber being formed with a positive charge (the harmful integrity model), which makes it difficult for the final NF S_1_′ to form a good morphology.

**Figure 3:**
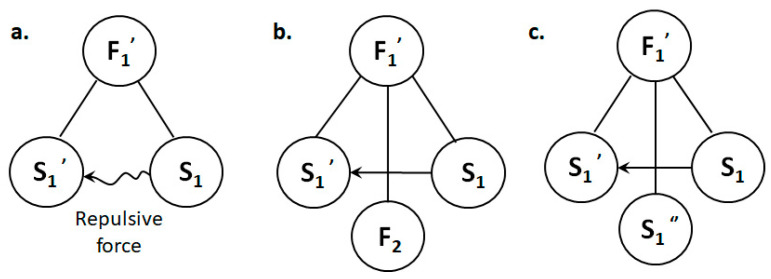
Methods of improving the substance–field model of the problem. (**a**) Hazardous substance–field model of the problem; (**b**) effective substance–field model after adding another field (F_2_); (**c**) effective substance–field model after improving S_1_ or S_1_′.

Therefore, the focus of the problem is how to solve the harmful complete model (Figure 3a), that is, how to prevent repulsion between the charged fiber and the fiber formed in the process [20].

### 2.2. Problem Solving

After analyzing the problem, the appropriate solution is selected from the standard solutions [21]. Based on their application process, five standard solutions to the destructive material field model can be applied to the harmful integrity model:

(1) F_2_ is used to offset the harmful effect;
(2) An improved S_1_ or/and S_2_ is introduced to eliminate the harmful effect;
(3) S_3_ is introduced to eliminate harmful effects;
(4) Harmful effects are eliminated;
(5) The magnetic influence is cut off.

For the complete harmful model shown in Figure 3a, the general solution provided by the substance–field analysis is to add another field, F_2_, to offset the effect of the original harmful field, as shown in Figure 3b, or add a third substance, S_3_, to prevent the harmful effect. Since adding another substance is the equivalent of adding extra items to the system, the same effect can be achieved by improving the current substance (S_1_ or S_1_′, represented by S_1_″), which can better meet the requirements of the IFR in innovative design, as shown in Figure 3c.

All of the standard solutions above may be effective in solving this problem. In this paper, F_2_ was first used as an example to solve the problem [22]. The corresponding substance–field model is shown in Figure 3b. Some scholars have counteracted charge repulsion by introducing external forces. For example, Sun et al. [8] were the first to prove the feasibility of attracting charged fibers with objects using heterogeneous charges through their experiments, thus neutralizing repulsion between fibers so that fibers could form an ideal 3D fiber stack. Then, they designed a physical device. The conversion of 2D film into 3D fiber stacks on a collector can be realized using an electrostatic generator. The 2D nonwoven pad on the collector is connected to the cathode of an electrostatic generator with a copper wire and a copper clip to provide a negative charge for the nonwoven pad. A schematic diagram of the device is shown below. Then, by rapidly turning the crank of the electrostatic generator during the ES process, the fibers growing upward can be observed, successfully achieving the conversion of a 2D fiber film into a 3D stack.

**Figure 4:**
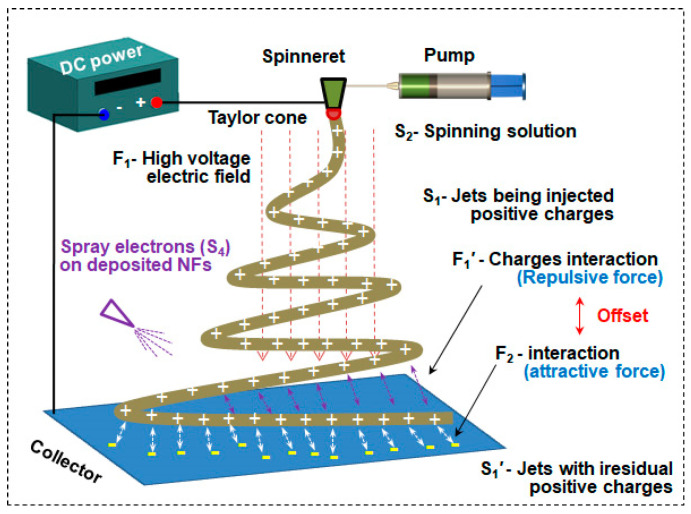
Schematic diagram of the device that negatively charges the receiving board.

**Figure 5:**
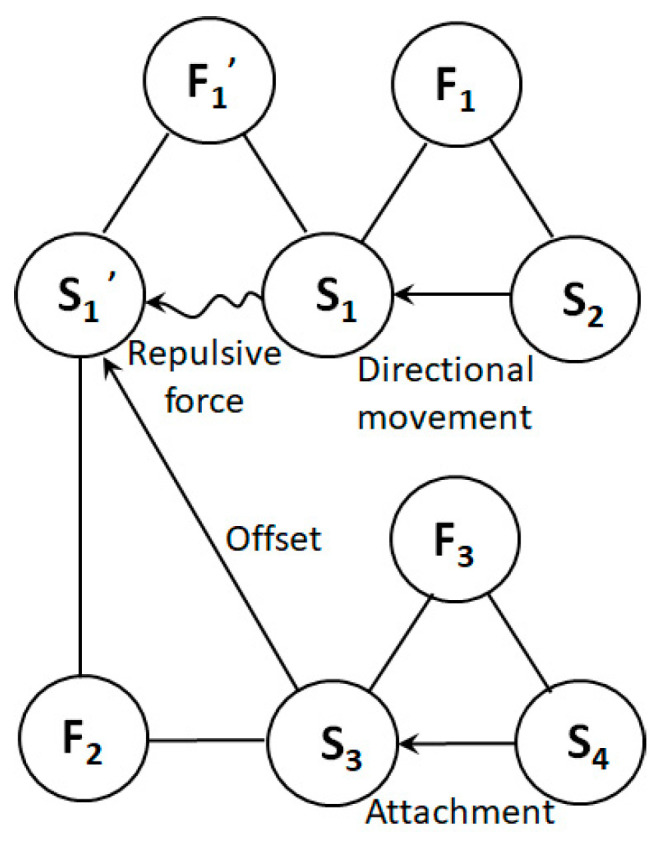
Substance–field model of the device that negatively charges the receiving board.

A substance–field model analysis of a problem involves the following steps:

1. Identification element. The model components can be identified when the system shown in Figure 4 is broken down. Materials: S2—spinning solution; S1—positively charged (+) filament being formed; S1′—positively charged (+) filament being formed; S4—negatively charged electron beam; S3—negatively charged receiving plate. Fields: F1—high voltage electric field; F1′—mutual repulsion between positive charges; F2—mutual attraction between positive and negative charges.

2. Build the model. Based on the organic combination of substances and fields, a complete substance–field model is constructed, as shown in Figure 5.

From the analysis of the substance–field model, the following can be seen:

(1) The positive charge in the spinning solution, S_2_, moves directionally under the action of the high-pressure electric field, F_1_, forming a positively charged fiber, S_1_, in the formation process;
(2) Under the interaction force, F_1_′, between charges, the formed fiber, S_1_′, has a repulsive effect on the formed fiber with a positive charge, S_1_′;
(3) The negatively charged electron, S_4_, is attached to the receiving board through the mechanical field, F_3_, making the receiving board, S_3_, negatively charged (-);
(4) The electric field, F_2_, attracted by the dissimilar charges acts on the formed and positively charged fiber, S_1_′, through the negatively charged receiving plate, S_3_, thus canceling the repulsive effect of S_1_ on S_1_′.

Problems in the substance–field model can then be analyzed. By constructing a substance–field model using Sun’s design, it can be seen that the design requires superfluous parts, S_4_ and F_3_, to introduce a new F_2_ to offset the harmful effect, which goes against the intent of the IFR principle in TRIZ theory. This is the equivalent of adding unnecessary structures and increasing the complexity of the whole device. Moreover, installing an electrostatic generator increases the cost of the whole device and worsens the energy loss rate. According to Sun’s article, the device’s receiver plate is placed in an open environment and connected to an electrostatic generator, reducing the safety of the system.

Tong et al. tried to solve this problem by combining two positively and negatively charged fibers to neutralize the residual charges. Polyvinyl alcohol and poly-racemic lactic acid were used as spinning materials for patterned NF mats with a consistent thickness, which proved the feasibility of the technology. A substance–field model analysis of the design follows.

**Figure 6:**
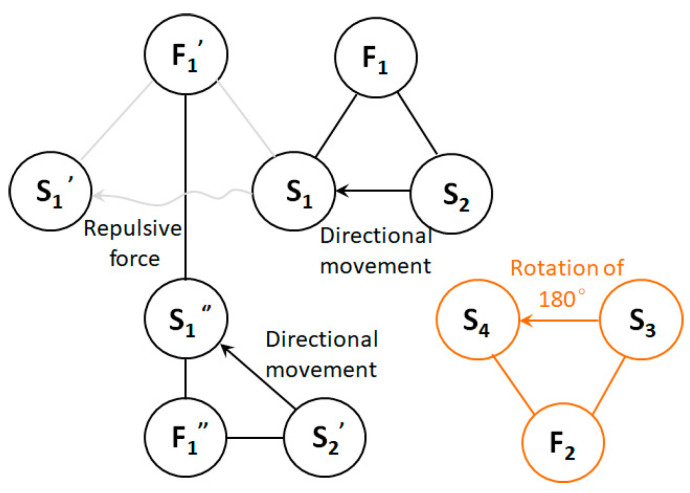
Substance–field model of a device that rotates the receiving plate to negatively charge the filaments.

(1) Identification element. Substances: S_2_—spinning solution with positive charge; S_2_′—spinning solution with negative charge; S_1_—fiber formed with positive charge (+); S_1_′—formed fiber with positive charge (+); S_1_″—improved fiber with negative charge (-); S_3_—disk base; S_4_—receiving plate. Fields: F_1_—positive high-voltage electric field; F_1_′—mutual repulsion between positive charges; F_1_″—negative high-voltage electric field; F_2_—mechanical field.
(2) Build the model. Based on the organic combination of substances and fields, a complete matter–field model can be constructed, as shown in Figure 6.

From the analysis of the substance–field model, the following can be seen:

(1) The positive charge in the spinning solution, S_2_, moves directionally under the action of the positive high-voltage electric field, F_1_, forming a positively charged fiber, S_1_, in the formation process. Note that this section of fiber forms on the receiving plate, S_4_, and becomes the positively charged fiber, S_1_′, that was already formed;
(2) The disk base, S_3_, causes the receiving plate, S_4_, to rotate 180° through the mechanical field, F_2_, of the motor, making the receiving plate, S_4_, face the spinning solution, S_2_′, under a negative charge;
(3) The negative charge in the spinning solution, S_2_′, moves directionally under the action of the negative high-pressure electric field, F_1_″, forming a negatively charged fiber, S_1_″. The repulsion of F_1_′ is eliminated by the improved S_1_″, and S_1_″ falls on S_4_ after forming;
(4) S_3_ causes S_4_ to rotate 180° in the opposite direction again so that S_4_ faces S_2_ with a positive charge. This cycle keeps repeating itself, spinning fibers that have no charge.

Problems in the substance–field model can then be analyzed. By constructing a substance–field model based on Tong’s design, it is not difficult to see that another substance–field model, unrelated to the original electric field, is introduced to solve the original problem. The design is redundant, which may cause new physical conflicts during the use of the equipment.

In addition, from the perspective of the equipment used after concretely implementing the design, this adds another set of injection equipment, unlike the original high-voltage spinning principle. This increases the complexity of the device, causes it to be too large, or increases its area. Moreover, the receiving plate has four areas, but only two areas work; thus, the collection efficiency is low, and motor energy is consumed to rotate the receiving plate, leading to unnecessary energy loss.

### 2.3. Device Improvement of ES

Although the current designs solve the problem related to difficulties in preparing 3D patterned fiber films caused by charge repulsion from different angles, through the above analysis, it can be seen that there are still problems in these designs. To solve the problems more efficiently and succinctly, IFR theory is used here to analyze the essence of the obstacles and available resources:

(1) What is the ultimate purpose of the design?

In the process of high-pressure electrostatic spinning, the fibers formed and the fibers already formed do not repel each other.

(2) What is the IFR?

There is no interaction between the fibers that are being formed and the fibers that are already formed.

(3) What are the barriers to achieving the IFR?

DC power spinning gives the fibers the same charge.

(4) What is the result of this problem?

The filaments repel each other, so it is difficult to prepare patterned fiber films with consistent thickness.

(5) What are the conditions for the absence of this problem?

There is no charge between the fibers or their charges are dissimilar.

(6) What are the resources available to create these conditions?

A high-voltage power supply, a propulsion unit, polymer material, spinning solvent, a container, a receiving plate and a ground.

**Figure 7:**
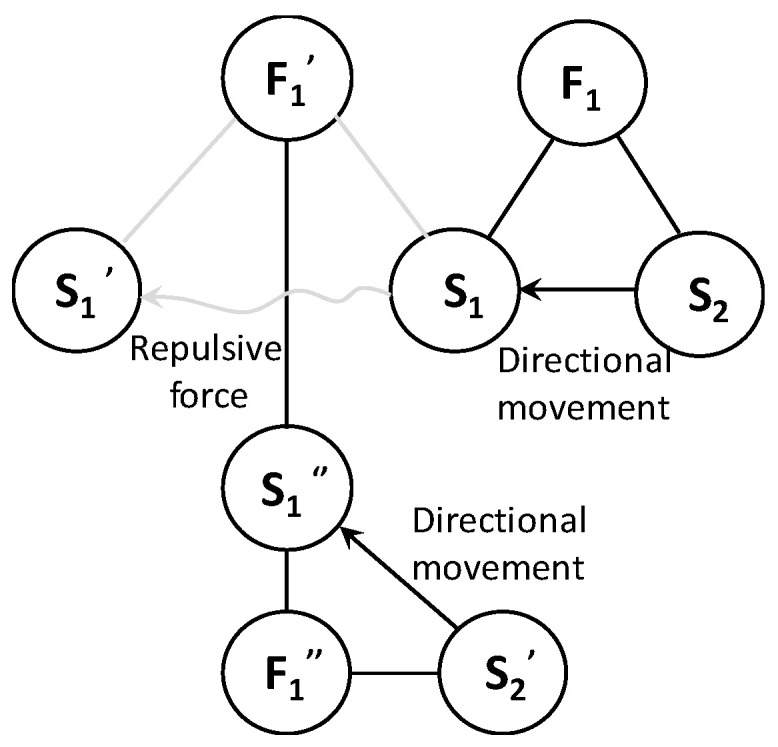
Substance–field model of the optimized design.

Using the “What resources were available when creating these conditions” prompt in IFR, one can see that other resources have been added to the existing designs [23]. Taking the restriction condition of not expanding resources as an innovation and combining this with the substance–field model of the current design, we can see that Tong’s substance–field model is complete. If the design can be improved on the basis of its substance–field model, there is no need to introduce other energy fields, which can solve the problems with the current device to the greatest extent. That is, the substance–field model of the optimized design should reflect the one shown in Figure 7, and the energy of the negative high-voltage electric field (F_1_″) should be provided by existing resources.

**Figure 8:**
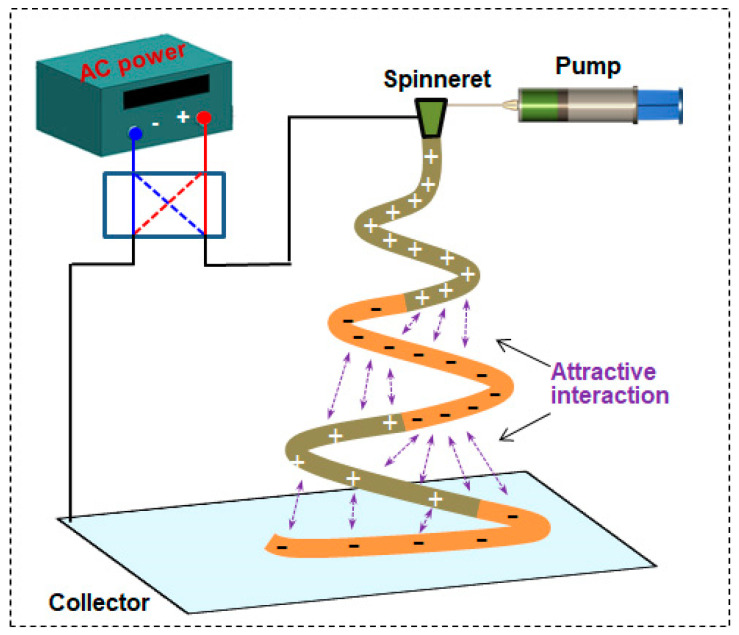
Schematic diagram of a device that uses low-frequency AC high voltage.

After this analysis using IFR theory, it can be concluded that technical conflicts prevent the existing solutions from solving the “removal of residual charge” problem. Therefore, the technical conflict resolution theory in TRIZ is used here to provide guidance in efficiently and feasibly resolving this contradiction. Thus, 39 general technical parameters and classical conflict matrices can be used to analyze the existing solution, and 40 invention principles can be used to optimize the solution [24]:

(1) Define the technical conflict. Eliminate the repulsive effect of electric field force between the fibers being formed and the fibers already formed; “repulsive force” is the parameter to be improved. In both of the above designs, there are structures that could not be added, and extra power is even needed, which creates useless work for the system and consumes energy; therefore, the deterioration parameter is “waste of energy”.
(2) Query the conflict matrix. “Repulsive force” corresponds to the general technical parameter “10—force”; “waste of energy” corresponds to the general engineering parameter “22—energy loss”. After querying the TRIZ theory conflict matrix table, two recommended invention principles were obtained: 14 (curved) and 15 (dynamic characteristics).
(3) Apply the invention principle. In combination with the IFR, a solution was proposed: Principle 15 (dynamic characteristics) was selected to adjust the performance of an object or environment to achieve an optimal state in all stages of work. The DC power was changed to a low-frequency AC power supply, and the operation mode of the object that belongs to the system was adjusted so that a single current was passed to the polymer from the variable alternating current power supply (AC power). In the spinning process, a periodic change in current provides alternating intervals of dissimilar charges for the fibers, and they neutralize each other, thus eliminating or weakening the repulsive effect and obtaining a patterned fiber film with a consistent thickness. The optimized solution not only cancels the repulsive force but also solves the problem of worsening technical conflict: there is no extra structure or device added to the system, reducing the energy loss. The improved device is shown in Figure 8. The AC frequency can be adjusted based on the controllability of the equipment and the specific spinning situation, generally set to 1–5 Hz. The AC frequency not only improves the controllability of the equipment but also matches the spinning frequency.

The optimized design also meets the evaluation criteria of the IFR system in TRIZ theory; that is, it completes the required functions with as little structure as possible. Compared with the DC power required by the original device, the AC current has a lower cost, greater versatility and universality, which is conducive to the wide use of spinning equipment. In general, this design helps the system realize a maximum degree of self-service with a minimum degree of change, which is closer to the ideal solution.

## 3. Experiment

### 3.1. Materials and Methods

Polystyrene-co-maleic anhydride (PSMA, Mw = 170 kDa, maleicanhydride content = 14.8 wt%), AgNO_3_ solution and dopamine hydrochloride (DA) aqueous solution (1 mg/mL) were received from Shanghai Zhaocheng Scientific Development Corp. (Shanghai, China). Dimethylformamide (DMF) solvents and other chemicals were analytical grade and received from Changzheng Regents Company (Chengdu, China) unless indicated otherwise. Voltage contactors (CKG4-12) were purchased from Bipu Electronic Co. (Shanghai, China), and other auxiliary electronics were purchased from YouXin Electronic Co. (Shenzhen, China).

Figure 8 illustrates the setup of an alternating ES apparatus, which comprised an AC power source, a voltage contactor, a spinneret, a pumping device and a collector. The two electrodes on the AC power source were connected to the voltage contactor, which conducted voltage between the spinneret and collector at a given frequency. The voltage and distance were set as 16 kV and 16 cm; the electric field intensity between the spinneret and collector was 1 kV/cm.

In total, 2 g of PSMA was dissolved in 8 mL of DMF and stirred for 5 h at 80 °C. Then, pure PSMA solution was loaded into the pumping device, and the feeding speed was set as 0.5 mL/h. ES was performed on the suspensions for 15 min; NFs were obtained on the collector; and the thickness of the NFs was recorded using an optical stereomicroscope (Zeiss Axio Lab A1, Oberkochen, Germany) [25]. The charges in the fibrous mats were measured with a programmable electrometer (Keithley6514, Cleveland, OH, USA), and the data were collected and recorded via computer-controlled measurement software written in LabVIEW [26].

The fibrous morphology was observed using a scanning electron microscope (SEM, FEI Quanta 200, Philips, The Netherlands) equipped with a field-emission gun and a Robinson detector. The density of the material, “ρ”, was provided by the supplier, and the mass, “m”, and the volume, “v”, of the NFs were measured with an electronic balance (Mettler Toledo XPR2U/AC, Zurich, Switzerland) and a vernier caliper (Deli, China). The porosity of the NFs was calculated via the following formula: [1 − (m/ρ) ÷ v] × 100% [27,28]. Image J was utilized to measure the average diameter by randomly picking 100 NFs from the SEM images [29]. The surface potentials of the NFs were measured using an electrostatic voltmeter (Isoprobe Model 279, New York, NY, USA).

### 3.2. Results and Discussion

**Figure 9:**
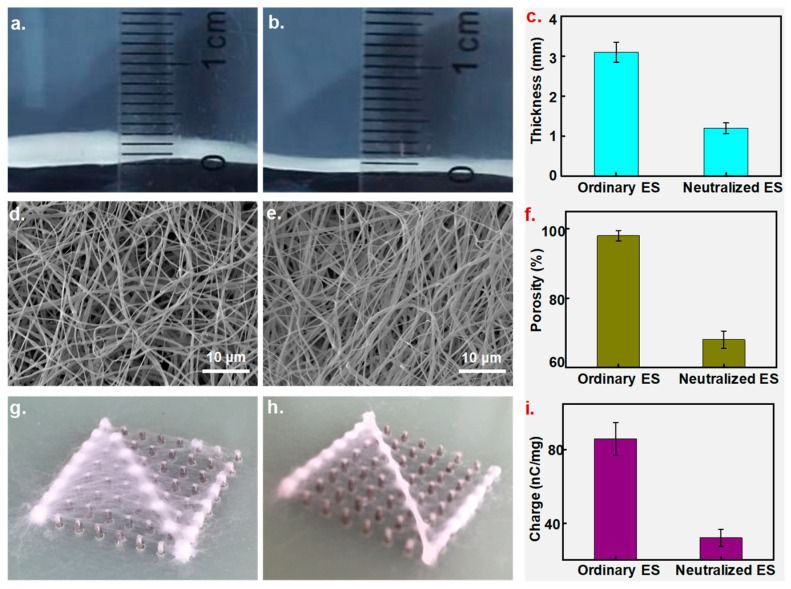
Characterization of NFs prepared using ordinary and neutralized ES. (**a**–**c**) Thickness of NFs in images and a statistical chart. (**d**–**f**) Thickness of NFs in SEM images and a statistical chart. (**g**,**h**) Direct-writing-patterned NFs created using ordinary and neutralized ES. (**i**) Residual charges within NFs collected using the two ES methods.

The collecting board comprised an array of 8 × 8 metal pillars, and all pillars could be programmed individually to construct a patterned conducting area. Figure 9a,b show the visual images of the collected NFs using ordinary and neutralized ES (NES) processes. Ordinary ES produced NFs with a thickness of around 3.1 mm, which was larger than that of neutralized ES NFs of 1.2 mm (Figure 9c). The thickness and depositing range of the NFs were determined based on the ES time and distance. Theoretically, under the same circumstances, NFs with thinner and more similar thicknesses than usual (required for denser fibrous mats) could be collected by using NES [30]. Figure 9d,e show SEM images of NFs distributed uniformly with a diameter of around 900 nm, prepared using the two methods; however, NFs created using NES appeared to be denser than ordinary ones. Statistics indicated that NFs created using ES had 98% porosity, which was 1/3 higher than that of those created using NES (61%, shown in Figure 9f). It seems that the NES technique significantly reduced repulsion effects and produced tight NFs. In further steps, two ES methods were applied to directly write the letter “Z”. As shown in Figure 9g, most NFs were deposited on the set path and formed a fibrous letter “Z”; however, quite a few dropped off the writing path and collected into a blurry, fibrous letter, probably because of severe repulsion as residual charges accumulated on the collector. Conversely, NES injected opposite charges in NFs, and the residual charges were neutralized, removing the expelling force and generating a patterned, fibrous letter with a clear boundary, as shown in Figure 9h. Residual charges in the NFs were recorded and displayed, as shown in Figure 9i; neutralization reduced the residual charges dramatically from 86 nC/mg to 32 nC/mg, and charge elimination weakened the repulsion effects dramatically, fabricating a patterned fiber.

To confirm the neutralization effects, an insulated polyethylene (PE) film was placed over the collector, and partial pillars were directed to form a pattern.

**Figure 10:**
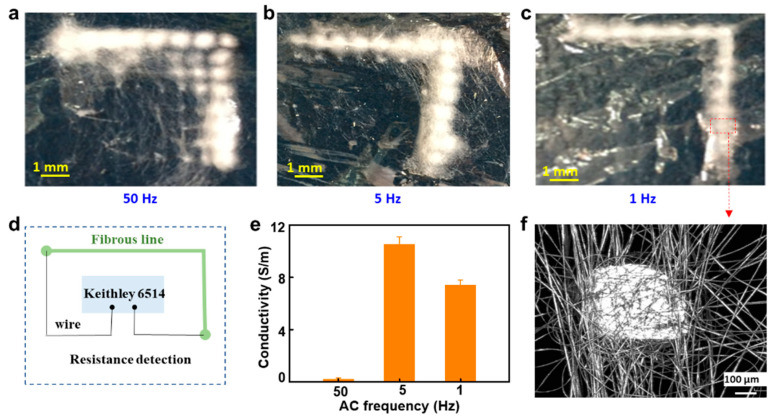
(**a**–**c**) Patterned NFs prepared using neutralized ES with different AC frequencies of 1, 5 and 50 Hz. (**d**) Resistance detection schematic. (**e**) Conductivities of the patterned fibers. (**f**) Microgram of NFs at 1 Hz AC.

In total, 1 mL of AgNO_3_ solution (5 mg/mL) was added to 10 mL of PSMA solution, and the mixture was stirred for one hour. Then, 1 mL of DA solution was added to obtain Ag nanoparticles after a 40 min reduction. Patterned NFs fabricated using different AC frequencies were characterized using an optical microscope, as shown in Figure 10a–c. The results demonstrated that low frequencies can produce more uniform patterned fibrous mats with high-frequency AC. NFs with 1 to 5 Hz AC exhibited better pattern continuity than those with 50 Hz. As PE film separated the NFs and conducting pillars, residual charges on the NFs had difficulty being conducted through the collector; therefore, the residual charge accumulation and NF repulsion effects were amplified. However, NES can neutralize most residual charges and reduce the repulsion effects, resulting in clear, fibrous patterns.

The conductivity of the patterned fibers was detected using a Keithley 6514 to demonstrate their continuity in further steps, as shown in Figure 10d. The conductivities of different AC frequencies are shown in Figure 10e; high-frequency AC had poor neutralization effects and only a few fibers were deposited between some of the pillars, which induced a low conductivity value of 0.2 S/m in the entire fibrous line. These results agree with the optical view in Figure 10a; there are several break gaps between the pillars. Patterned fibers with a low frequency of between 1 and 5 Hz showed better conductivity at 7.5 and 10.4 S/m. A microscopic view of the fibrous lines on a local pillar is shown in Figure 10f, which illustrates the possibility of simply and conveniently fabricating flexible electric lines and other electronics.

## 4. Conclusions

In conclusion, a novel NES technique was designed using TRIZ theory. AC power was applied to a spinneret, and polymer jets were partially injected with positive or negative charges. Residual charges in the NFs with different polarities were covered one after another, resulting in weakened repulsion with most residual charges neutralized. Finally, patterned NFs with clear boundaries were successfully obtained. This innovation improved the quality and efficiency of the patterned NFs and opened up new possible application fields.

This direct writing technique, derived from conventional ES, could be used to produce ultrafine NFs with desirable patterns, and this, in turn, could provide new ways to fabricate flexible circuits in customized patterns with conductive NFs, with different circuits only requiring a modified control program. Furthermore, a collector with an array composed of more tiny pillars could be prepared by using micro-electromechanical systems (MEMSs) to obtain more sophisticated fibrous circuit patterns. Except for AC frequency, other parameters in the ES process should be investigated. To achieve industrialization, more effort should be put into optimizing ES techniques to obtain high-quality patterned and functionalized NFs.

## Data Availability

Not applicable.

## References

[1] Xue J., Wu T., Dai Y., Xia Y. (2019). Electrospinning and Electrospun Nanofibers: Methods, Materials, and Applications. Chem. Rev..

[2] Yan T., Wu Y., Pan Z. (2021). Anisotropy of resistance-type strain sensing networks based on aligned carbon nanofiber membrane. J. Mater. Sci..

[3] Formhals A. (1934). Process and Apparatus for Preparing Artificial Threads. U.S. Patent.

[4] Ding J.X., Zhang J., Li J.N., Li D., Xiao C., Xiao H., Yang H., Zhuang X., Chen X. (2019). Electrospun polymer biomaterials. Prog. Polym. Sci..

[5] Islam M.S., Ang B.C., Andriyana A., Afifi A.M. (2019). A review on fabrication of nanofibers via electrospinning and their applications. SN Appl. Sci..

[6] Kenry C.T.L. (2017). Nanofiber technology: Current status and emerging developments. Prog. Polym. Sci..

[7] Tong H.W., Wang M. (2013). A novel technique for the fabrication of 3D nanofibrous scaffolds using simultaneous positive voltage ES and negative voltage electrospinning. Mater. Lett..

[8] Sun B., Long Y.Z., Yu F., Li M.M., Zhang H.D., Li W.J., Xu T.X. (2012). Self-assembly of a three-dimensional fibrous polymer sponge by electrospinning. Nanoscale.

[9] Li Y., Li W.Q. (2013). Method to Creative Design.

[10] Stepanyan R., Subbotin A.V., Cuperus L., Boonen P., Dorschu M., Oosterlinck F., Bulters M.J. (2016). Nanofiber diameter in electrospinning of polymer solutions: Model and experiment. Polymer.

[11] He X.X., Zheng J., Yu G.F., You M.H., Yu M., Ning X., Long Y.Z. (2017). Near-field electrospinning: Progress and applications. J. Phys. Chem. C.

[12] Deshawar D., Chokshi P. (2017). Stability analysis of an electrospinning jet of polymeric fluids. Polymer.

[13] Andreas G., Wendorff J.H. (2007). Electrospinning: A fascinating method for the preparation of ultrathin fibers. Angew. Chem. Int. Ed..

[14] Cheng J., Jun Y., Qin J., Lee S.H. (2017). Electrospinning versus microfluidic spinning of functional fibers for biomedical applications. Biomaterials.

[15] Zhao S., Zhou Q., Long Y.Z., Sun G.H., Zhang Y. (2013). Nanofibrous patterns by direct electrospinning of nanofibers onto topographically structured non-conductive substrates. Nanoscale.

[16] Chen Y., Zhao M., Xie Y., Zhang Z. (2015). A new model of conceptual design based on Scientific Ontology and intentionality theory. Part II: The process model. Des. Stud..

[17] Feng P.E., Chen Y., Zhang S., Pang S. (2002). Conceptual design based on product genetics. Chin. J. Mech. Eng..

[18] Li W.Q., Li Y., Wang J., Liu X. (2010). The process model to aid innovation of products conceptual design. Expert Syst. Appl..

[19] Zheng H., Feng Y.X., Gao Y.C., Tan J. (2018). The solving process of conceptual design for complex product based on performance evolution. Chin. J. Mech. Eng..

[20] Liu X.M., Tan R.H., Yao L.G. (2008). Application research on integrated process model for the conceptual design of product innovation. Chin. J. Mech. Eng..

[21] Dorst K., Cross N. (2001). Creativity in the design process: Co-evolution of problem-solution. Des. Stud..

[22] Qian H., Li Y., Tao Y., Liu H. (2020). Triple-helix structured model based on problem-knowledge-solution co-evolution for innovative product design process. Chin. J. Mech. Eng..

[23] Wang P., Su J.N., Hu C.B., Zhang S.T. (2013). Research on Product Identity Design Based on Kansei Image in Mechanic Equipment. Adv. Mater. Res..

[24] Gero J.S., Kannengiesser U. (2004). The situated function–behaviour–structure framework. Des. Stud..

[25] Jiang S.H., Chen Y.M., Duan G., Mei C., Greiner A., Agarwal S. (2018). Electrospun nanofiber reinforced composites: A review. Polym. Chem..

[26] Yi F., Wang X., Niu S., Li S., Yin Y., Dai K., Zhang G., Lin L., Wen Z., Guo H. (2016). A highly shape-adaptive, stretchable design based on conductive liquid for energy harvesting and self-powered biomechanical monitoring. Sci. Adv..

[27] Kim K., Dean D., Mikos A.G., Fisher J.P. (2009). Effect of Initial Cell Seeding Density on Early Osteogenic Signal Expression of Rat Bone Marrow Stromal Cells Cultured on Cross-Linked Poly (propylene fumarate) Disks. Biomacromolecules.

[28] Vitale A., Massaglia G., Chiodoni A., Bongiovanni R., Pirri C.F., Quaglio M. (2019). Tuning Porosity and Functionality of Electrospun Rubber Nanofiber Mats by Photo-Crosslinking. ACS Appl. Mater. Interfaces.

[29] Liu Q.J., Wu Q., Xie S.Z., Zhao L., Chen Z., Ding Z., Li X. (2019). Uniform field electrospinning for 3D printing of fibrous configurations as strain sensors. Nanotechnology.

[30] Tripatanasuwan S., Zhong Z., Reneker D.H. (2007). Effect of evaporation and solidification of the charged jet in electrospinning of poly(ethylene oxide) aqueous solution. Polymer.

